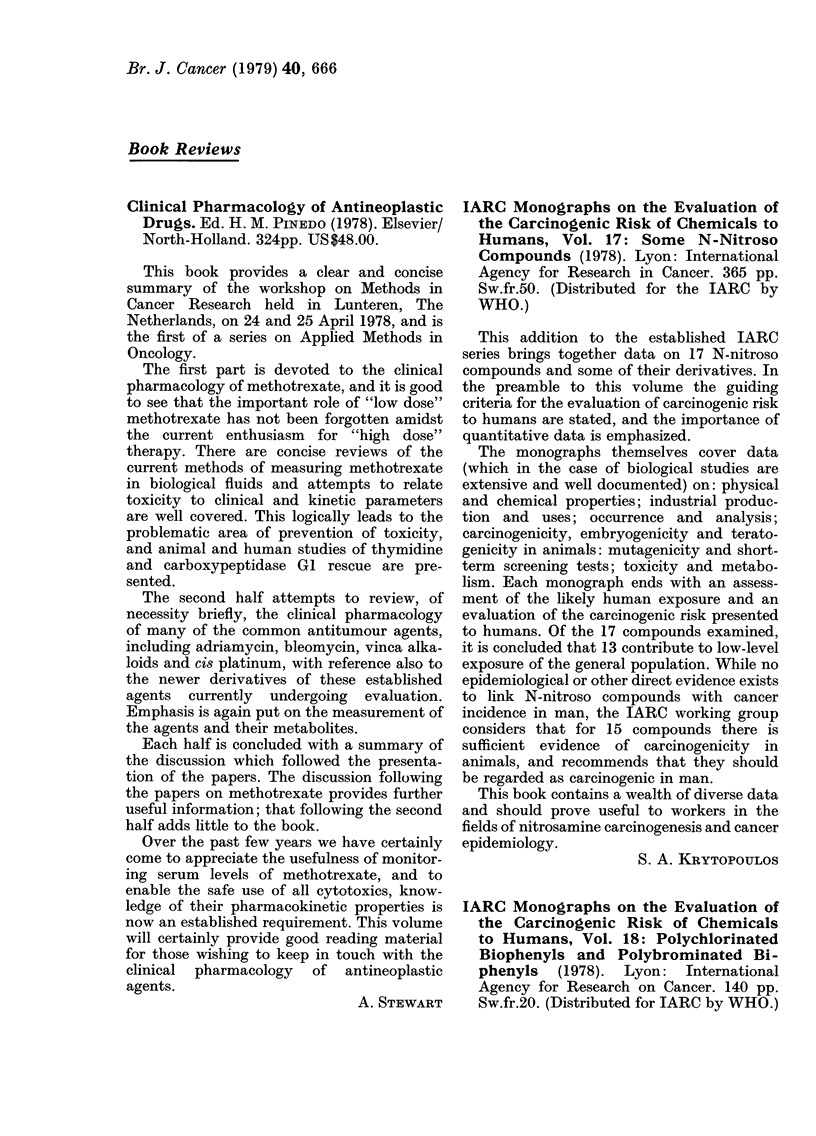# Clinical Pharmacology of Antineoplastic Drugs

**Published:** 1979-10

**Authors:** A. Stewart


### *Book Reviews*

**Clinical Pharmacology of Antineoplastic Drugs.** Ed. H. M. PINEDO (1978). Elsevier/North-Holland. 324pp. US$48.00.

This book provides a clear and concise summary of the workshop on Methods in Cancer Research held in Lunteren, The Netherlands, on 24 and 25 April 1978, and is the first of a series on Applied Methods in Oncology.

The first part is devoted to the clinical pharmacology of methotrexate, and it is good to see that the important role of "low dose" methotrexate has not been forgotten amidst the current enthusiasm for "high dose" therapy. There are concise reviews of the current methods of measuring methotrexate in biological fluids and attempts to relate toxicity to clinical and kinetic parameters are well covered. This logically leads to the problematic area of prevention of toxicity, and animal and human studies of thymidine and carboxypeptidase G1 rescue are presented.

The second half attempts to review, of necessity briefly, the clinical pharmacology of many of the common antitumour agents, including adriamycin, bleomycin, vinca alkaloids and *cis* platinum, with reference also to the newer derivatives of these established agents currently undergoing evaluation. Emphasis is again put on the measurement of the agents and their metabolites.

Each half is concluded with a summary of the discussion which followed the presentation of the papers. The discussion following the papers on methotrexate provides further useful information; that following the second half adds little to the book.

Over the past few years we have certainly come to appreciate the usefulness of monitoring serum levels of methotrexate, and to enable the safe use of all cytotoxics, knowledge of their pharmacokinetic properties is now an established requirement. This volume will certainly provide good reading material for those wishing to keep in touch with the clinical pharmacology of antineoplastic agents.

A. STEWART